# Supplementary material for: A deep learning approach to programmable RNA switches
Source: Nat Commun. 2020 Oct 7;11:5057. doi: 10.1038/s41467-020-18677-1 (PMC7541447; doi:10.1038/s41467-020-18677-1)
Supplement: Supplementary file 1 — Supplementary Information [file 41467_2020_18677_MOESM1_ESM.pdf]

Supplementary Information for

A Deep Learning Approach to Programmable RNA Switches

Nicolaas M. Angenent-Mari, Alexander S. Garruss, Luis R. Soenksen,
George Church, and James J. Collins

Correspondence to: jimjc@mit.edu

This PDF file includes:

Supplementary Figures (S1 to S16)
Supplementary Tables (S1 to S5)

A

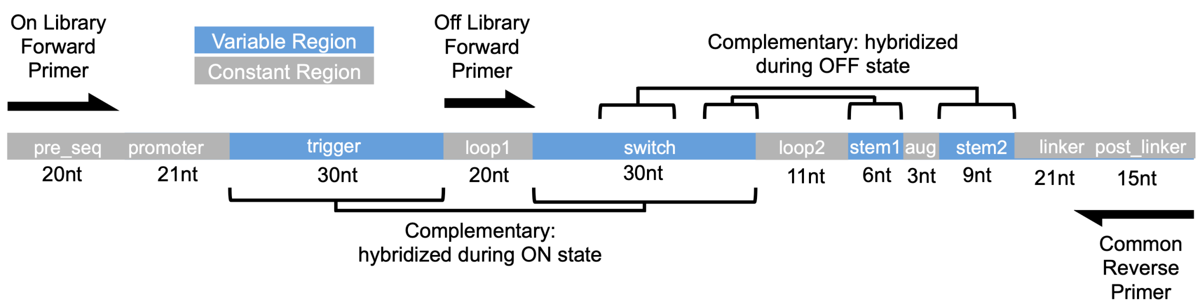

Constant Region Sequences

pre_seq: CTCTGGGCTAACTGTCGCGC
promoter: TAATACGACTCACTATAGGG
loop1: AACCAAACACACAAACGCAC
loop2: AACAGAGGAGA
linker: AACCTGGCGGCAGCGCAAAAG
post_linker: ATGCGTAAAGGAGAA

Primer Sequences (BsmBI in Blue, T7 Promoter in Green)

Common Reverse Primer: CGTCTCGTCTTCTCCTTTACGCATCTTTTG
On Forward Primer: CGTCTCGTGCTCTGGGCTAACTGTCGC
Off Forward Primer:
CGTCTCGTGCTCTGGGCTAACTGTCGCGCTAATACGACTCACTATAGGG
AACCAAACACACAAACGCAC

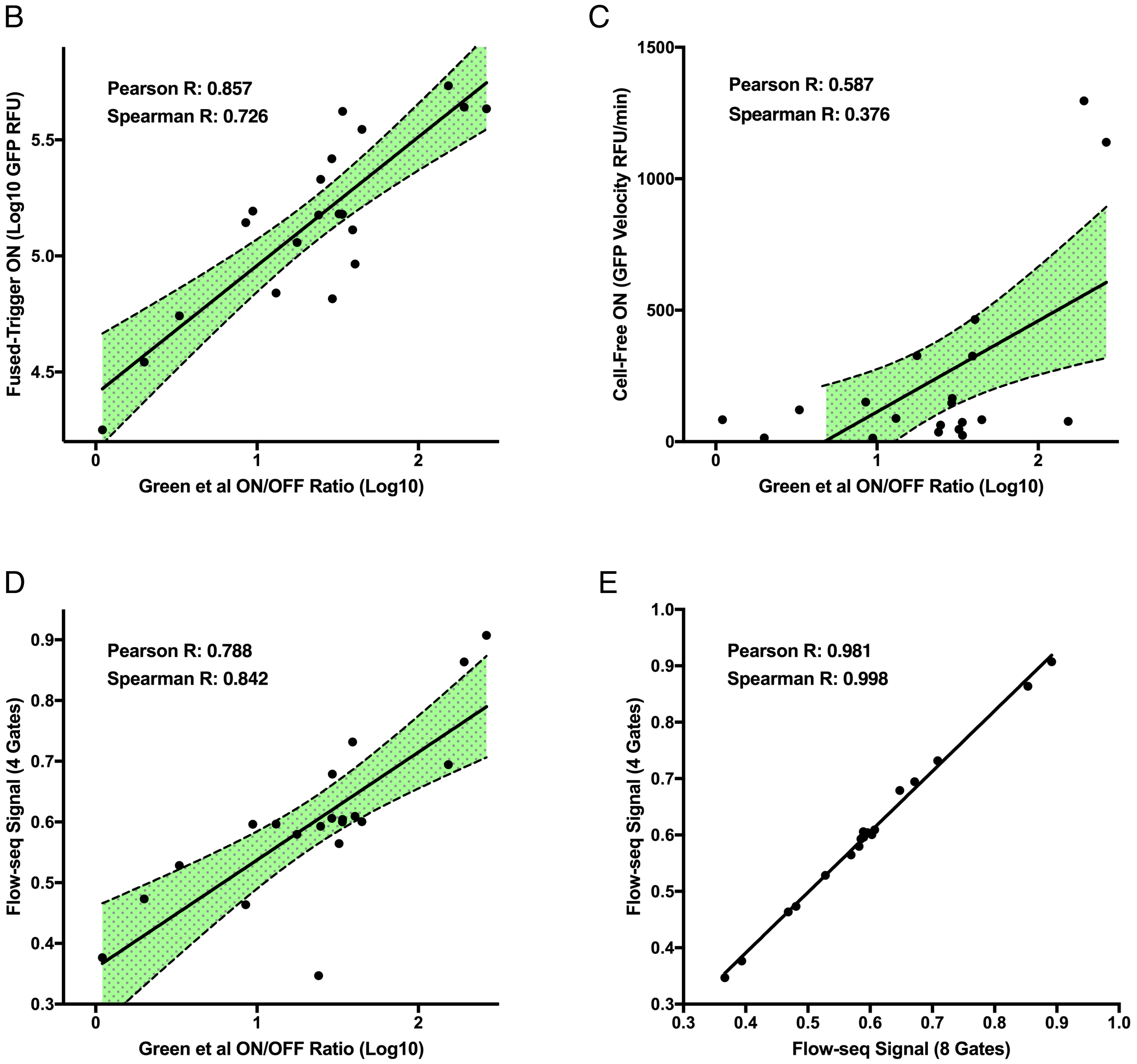

Fig. S1. Design and validation of the oligomer library. Individual toehold switch constructs within the library were synthesized from a pool of oligomers, and a representative panel of constructs was verified against a previously published dataset. (A) A schematic of the pooled library oligo used for the synthesis of our high-throughput toehold switch library. Distinct toehold construct regions include the *pre_seq* (plasmid backbone sequence), *promoter* (T7 promoter including GGG), *trigger* (toehold-unique), *switch* (complete toehold and ascending stem), *loop1* (region linking *trigger* to *switch*), *loop2* (main toehold switch hairpin loop containing the RBS), *stem1* (top half of descending stem), *atg* (start codon), *stem2* (bottom half of descending stem), *linker* (21nt sequence of unstructured amino acids), and *post_linker* (first 15nt of GFP). Further details can be found in Supplementary Table 4. The amplification primers for ON and OFF libraries (including the common reverse primer) are shown with black arrows. The ON/OFF ratios for a panel of 20 switches previously characterized in cells using unfused triggers by Green et al. (*1*) were compared against three new panels of equivalent switches prepared in this work: (B) the ON state GFP expression from toehold switches individually assayed in cells with fused triggers (Pearson R=0.857, Spearman R=0.726, error bands indicate 95% CI), (C) the ON state GFP expression from toehold switches individually assayed in a cell-free protein expression system (CFPS) with unfused triggers (Pearson R=0.587, Spearman R=0.376, error bands indicate 95% CI), and (D) the ON state GFP expression from toehold switches assayed with a flow-seq assay under sorting conditions identical to those used in producing our larger toehold switch dataset (Pearson R=0.788, Spearman R=0.842, error bands indicate 95% CI). (E) Flow-seq measurements were taken of the ON state of a panel of 20 toehold switches previously reported by Green et al. *(1)* using either four or eight sorting gates, and the agreement between the two methods was assessed using the Pearson and Spearman correlation coefficients (0.981 and 0.998, respectively, FACS data can be found in Supplementary Figure 2A). All source data are provided as a Source Data file. GFP= Green fluorescent protein, nt=nucleotide, RBS=Ribosome binding site, CI=Confidence Interval.

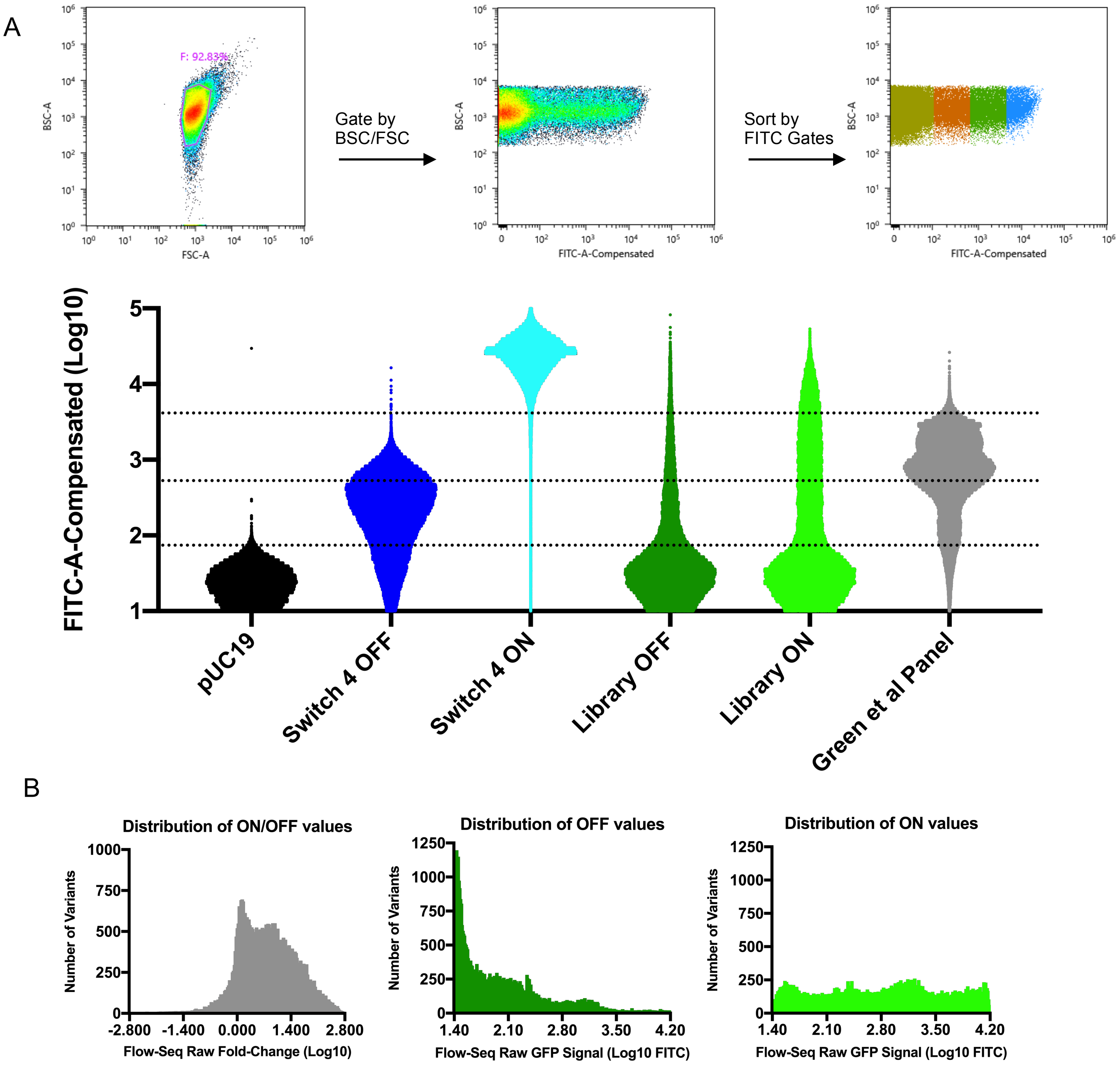

Fig. S2. Library FACS distributions and their empirically-derived sorting gates. To determine the boundaries of the sorting gates for our high-throughput toehold switch pipeline, we used Switch #4 from Green et al. *(1)* in ON and OFF conformations as a positive control, and a pUC19 plasmid lacking a GFP gene as a negative control. A pooled panel of twenty switches from Green et al. *(1)* was used to determine the optimal number of sorting gates. (A) The gating strategy for sorting IPTG-induced *E. coli* BL21-star cells by GFP fluorescence is shown (top), as well as FITC distribution plots from the three control conditions, the complete ON and OFF libraries, and the pooled panel of twenty switches from Green et al. *(1)*, with the boundaries of the four final sorting bins shown as dotted lines (bottom). (B) The resulting measurements obtained for ON, OFF, and ON/OFF using our flow-seq pipeline are shown as raw fluorescence values or raw fold change, rather than normalized to the range of [0,1] or [-1, 1] as in Figure 3. All source data are provided as a Source Data file.

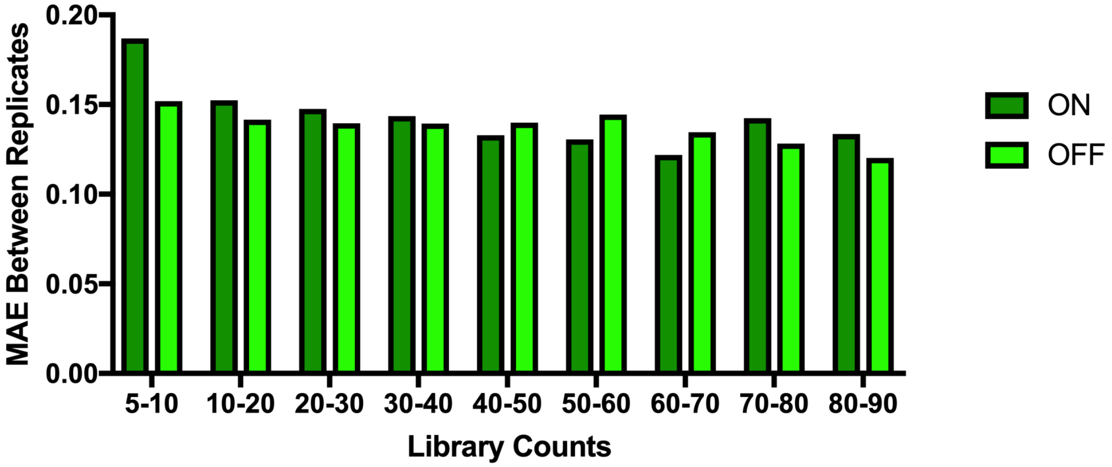

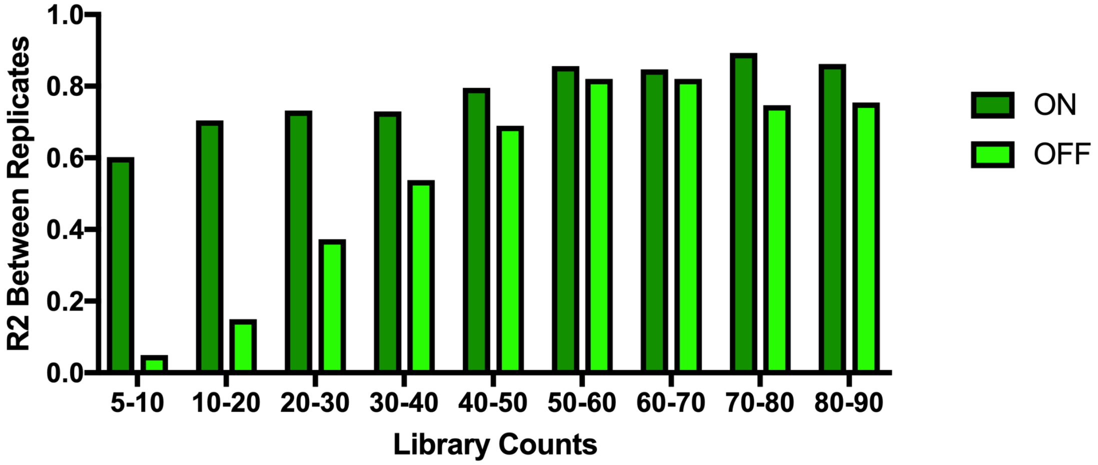

Fig. S3. The inter-replicate variability of our toehold switch libraries. For the same initial toehold library, we performed two replicates of the BL21 transformation process, followed by independent induction, sorting, and sequencing. Two metrics were used to compare the inter-replicate variability: the mean absolute error (MAE, top panel), and the R^2 correlation coefficient (bottom panel). Shown here are the MAE and R^2 values for ON and OFF measurements at different ranges of library count thresholds. All source data are provided as a Source Data file.

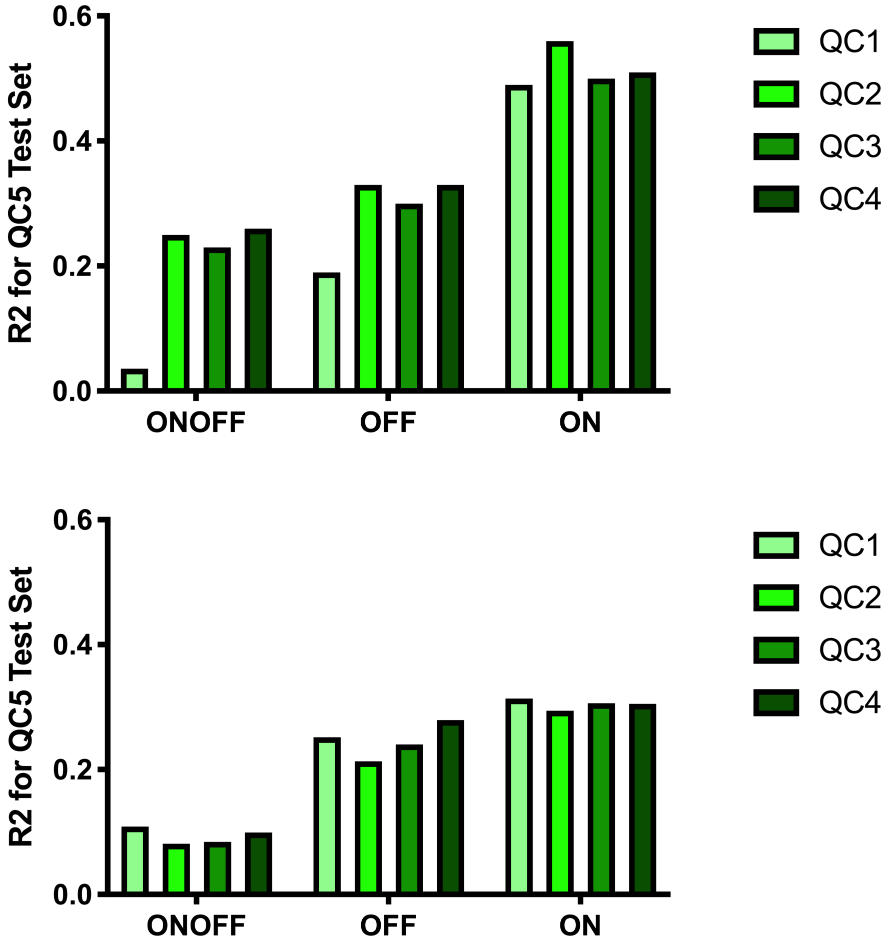

Fig. S4. The effect of QC level on MLP performance. The predictive power of our multilayer perceptron model was evaluated after training with datasets obtained from increasingly stringent quality control (QC) thresholds to determine an optimal balance between dataset size and quality. The most stringent quality control group (QC5) was withheld as a test set, and an MLP trained either on a one-hot representation of the toehold sequence (top panel) or 30 rational thermodynamic parameters (bottom panel) was given either QC1, QC2, QC3, or QC4 as training data. From the resulting test-prediction of QC5 values, we show the R^2 correlation metric between the predicted and experimental values. See Supplementary Table 1 for conditions for each QC level. All source data are provided as a Source Data file.

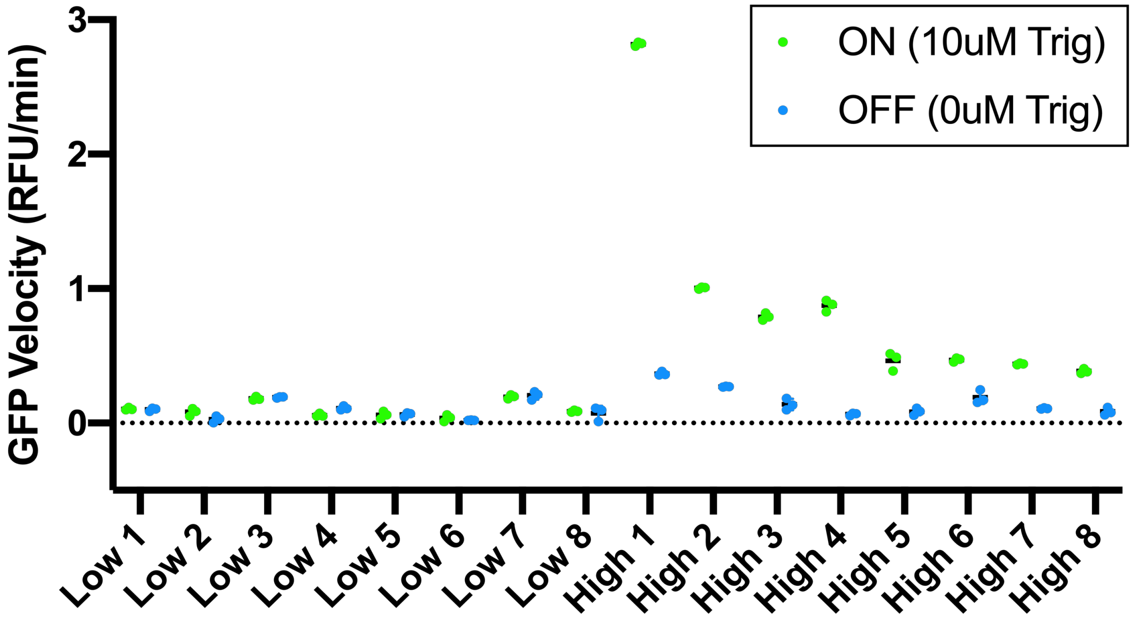

Fig. S5. Cell-free toehold switch validation. A panel of toeholds that showed either a low or high ON/OFF ratio as measured by our high-throughput flow-seq assay were individually cloned and assayed in a cell-free protein synthesis (CFPS) system. Dot plots for the timecourse velocities of GFP signal evolution are shown for the PURExpress CFPS reactions containing the 16 switches with or without their separately transcribed RNA triggers. The sequences and flow-seq assay results for these 16 switches can be found in Supplementary Table 2. Horizontal bars show the mean of n=3 biologically independent samples. All source data are provided as a Source Data file.

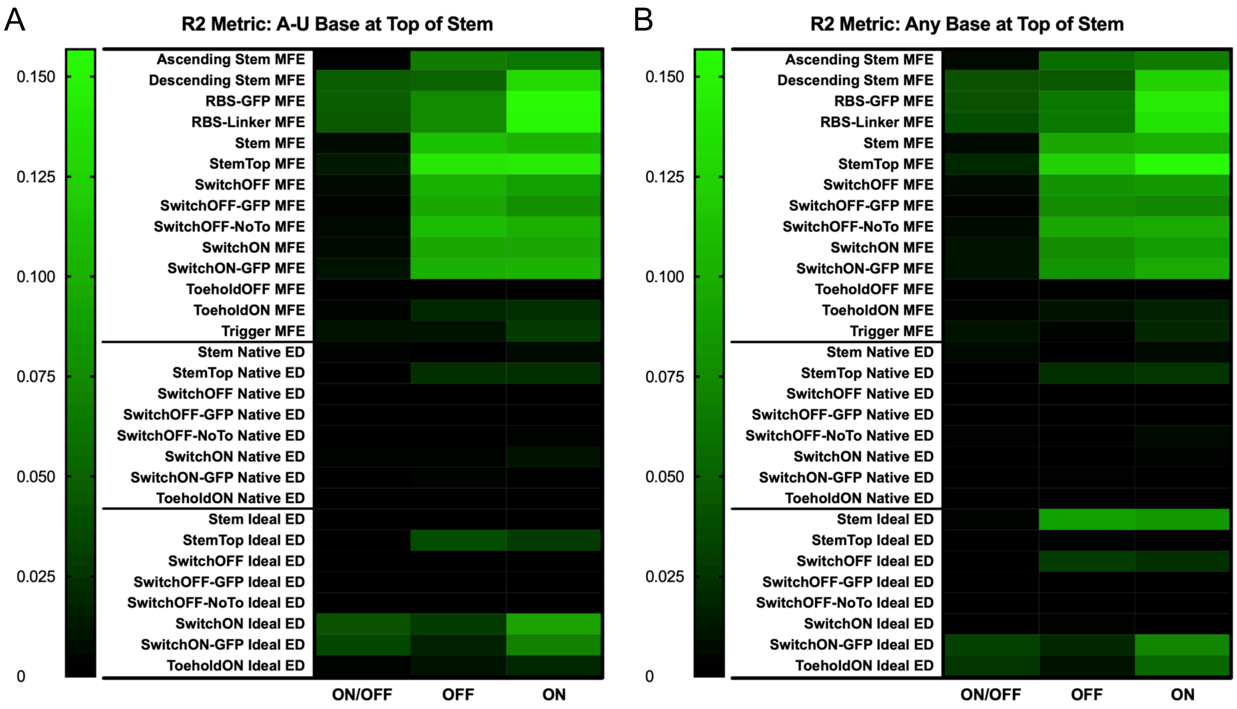

Fig. S6. Correlations between rational thermodynamic features and the toehold switch dataset, subsetted for A-U content. We analyzed the R^2 coefficients between 30 commonly used thermodynamic features and the ON, OFF, or ON/OFF measurements of variants in our high-throughput dataset. (A) R^2 coefficients for the subset of switches that contained only an A-U or U-A base pair at the top of the toehold switch stem (positions 79 and 91 in Supplementary Table 4). (B) R^2 coefficients for the entire set of switches, allowing for any base pair at the top of the toehold switch stem. Both R^2 value sets were compared to evaluate findings from Green et al. (*1*) where subsetting for switches with an A-U or U-A basepair at the top of the stem was sufficient to dramatically increase the predictive R^2 coefficient between thermodynamic features and measured ON/OFF. We found measurable differences between various thermodynamic features when subsetting for an A-U basepair at the top of the hairpin stem, particularly for those in the Ideal Ensemble Defect (ED) block. However, differences between the R^2 values in said subset and those obtained for other possible base-pairs were not statistically significant suggesting no overall increase in predictive value ($p>0.05$ for ON, OFF, and ON/OFF, two-tailed t-test). All source data are provided as a Source Data file

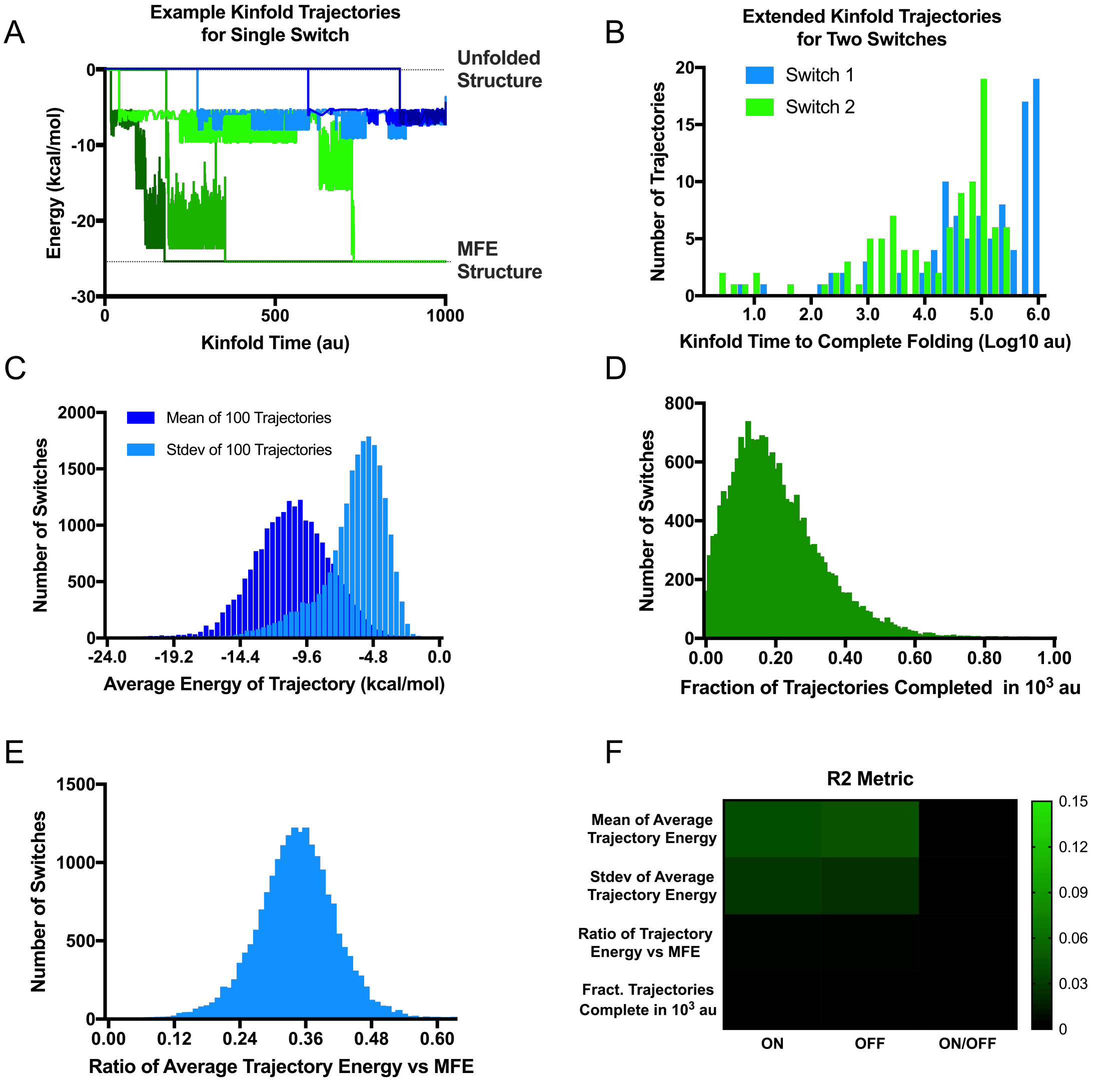

Fig. S7. Kinetic toehold switch folding analysis using Kinfold. Folding trajectories were run using the Kinfold package for the OFF-state switch sequence (positions 50-134nt in Supplementary Table 4). (A) For a single representative toehold switch, six example trajectories are shown. Trajectories in green reached the MFE structure within 10^3 arbitrary time units (au), while those in blue did not. (B) For two representative toehold switches, 100 trajectories were run for a maximum time of 10^6 au. Histograms of the time required for a trajectory to reach the MFE structure are shown. Most trajectories took longer than 10^3 au, compared to the Kinfold analyses in Borujeni et al. (2), where average trajectory times fell in the range of 10^1-10^3 au, and 10^4 au was the longest allowed trajectory time. (C,D,E,F) For each switch in the QC4 dataset (total 19,983 variants), 100 trajectories were run and the following measurements plotted: (C) histograms of the mean and negative standard deviation of the trajectories' average energy during the first 10^3 au, (D) the fraction of trajectories that completed folding of the MFE structure before 10^3 au, (E) the ratio of average trajectory energy to the minimum possible MFE energy, and (F) the R^2 correlation between the metrics in C,D,E and the empirical measurements in our toehold switch dataset. For comparison with previous rational features the heatmap axis is set similarly to Figure 3B. All source data are provided as a Source Data file.

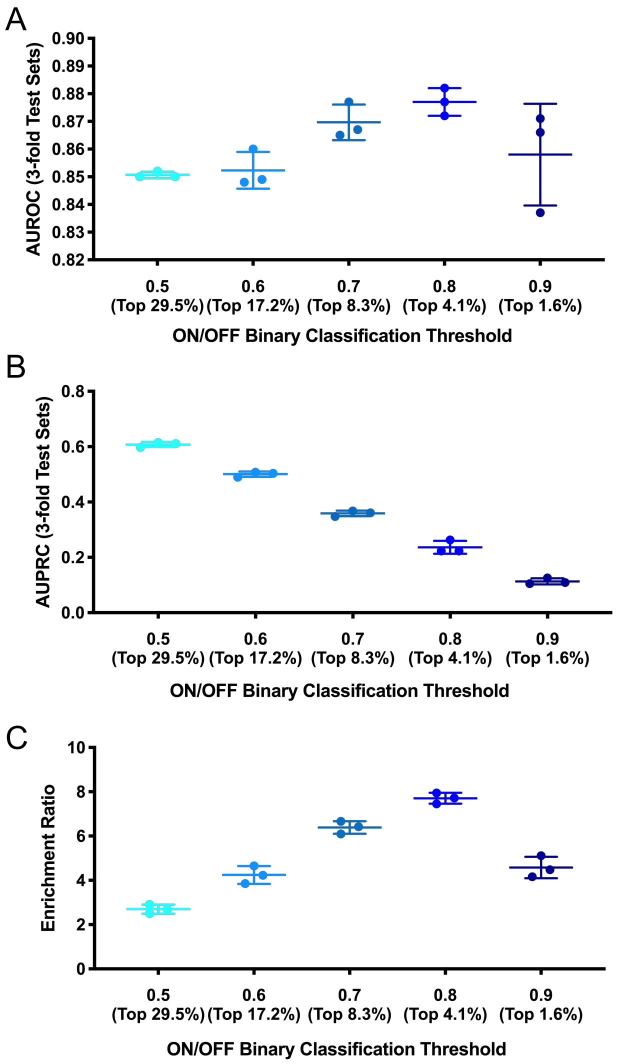

Fig. S8. Determination of the optimal ON/OFF binary classification cutoff threshold. AUC, P-R, and enrichment ratio analyses were used to determine the optimal cutoff threshold at which to binarize ON/OFF data for classification. We trained a standard MLP architecture on the one-hot sequence representation of the toehold switch at five different binarization thresholds, and compared the following performance metrics: (A) model AUROC results, (B) model AUPRC results, and (C) model enrichment ratio over random chance. The enrichment ratio is calculated as the fraction of true positive toehold switches returned by the model (i.e., the precision) divided by the fraction returned by random chance. The enrichment ratio was specifically calculated at the level of precision for which the recall returns one positive switch per 100, or approximately ten on average for a typical mRNA of length ~1000nt. The final threshold selected for all classification models in this study was 0.7 (or the top 8.3% of switches), balancing a high enrichment ratio with a practical degree of overall precision. Mean and standard deviation are shown for n=3 independently shuffled test sets. All source data are provided as a Source Data file.

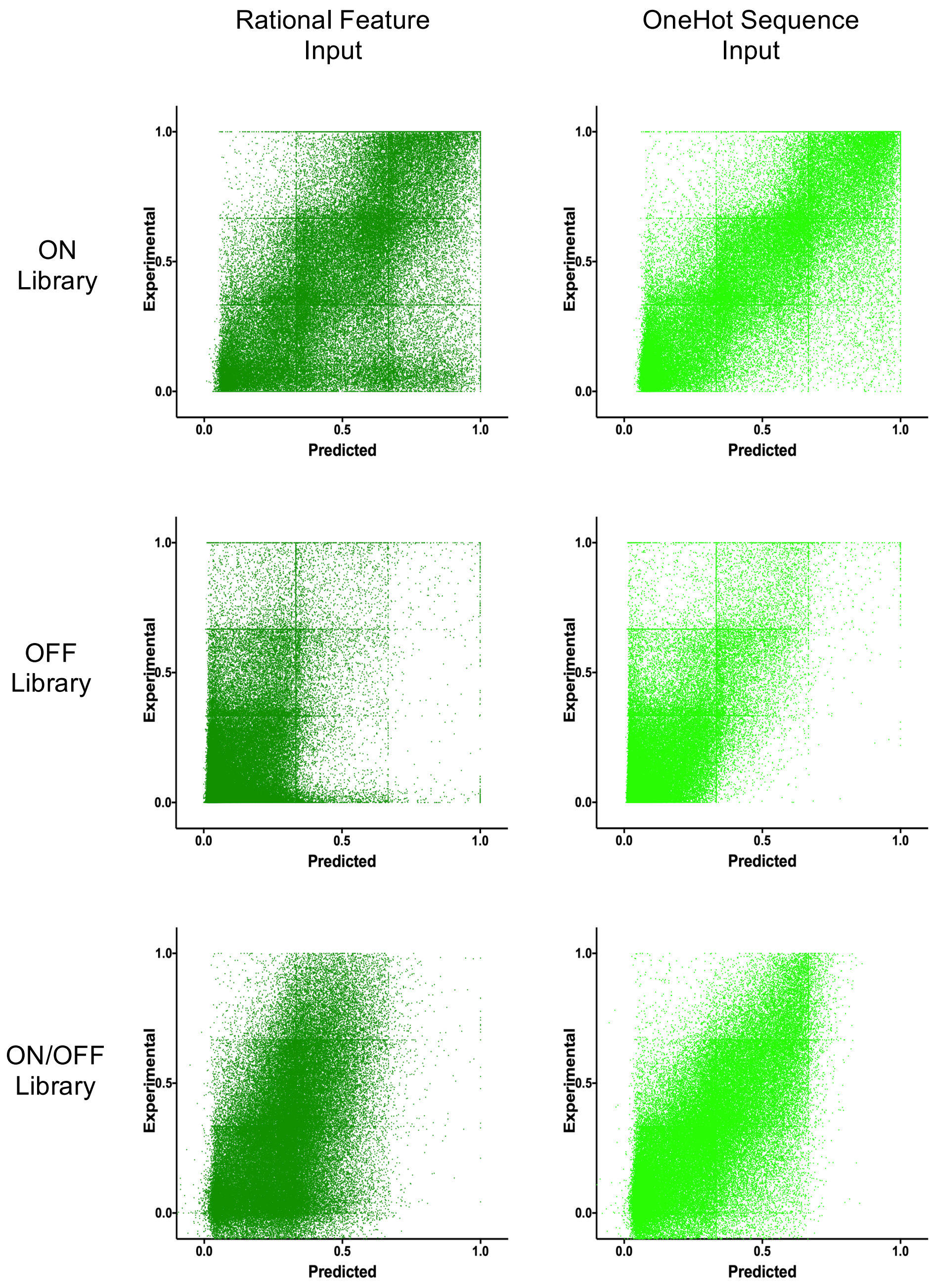

Fig. S9. Comparing our MLP predictions to our experimental results. Scatter plots of the predicted versus empirical values of our compiled test set are shown for ten-fold cross-validated MLP models trained with either the 30 pre-calculated rational thermodynamic features as inputs (left, dark green), or the toehold switch one-hot sequence representation as input (right, light green) for ON, OFF, and ON/OFF. The summary statistics are reported in Figure 3D,E. All source data are provided as a Source Data file.

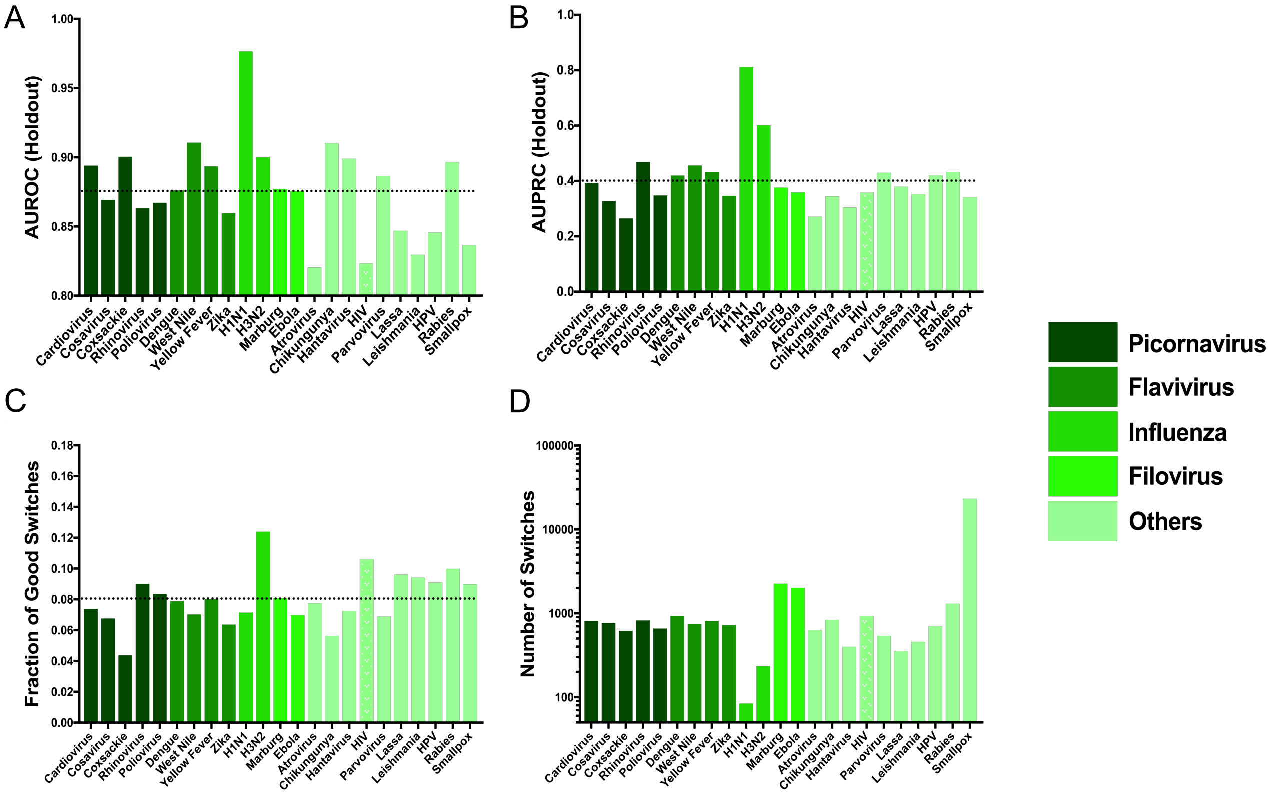

Fig. S10. Holdout validation of individual viral genomes. For each of the 23 pathogenic viruses tiled in our toehold switch dataset, every toehold switch targeting a given viral genome was withheld, and an MLP model was trained with the remaining sequences in the dataset using a one-hot sequence input representation classifying for ON/OFF ratio. The model performance was then evaluated on the switches of the withheld viral genoma as a test set. (A) Area under the receiver operating characteristic curves (AUROC) for holdout viral genomes. Dotted line denotes AUROC average across test samples. (B) Area under the precision-recall curves (AUPRC) for holdout viral genomes. Dotted line denotes AUPRC average across test samples. (C) Fraction of toehold switches in synthesized high-throughput library classified as high-performing for each virus type. Dotted line denotes average at 8%. (D) Total number of toehold switches synthesized for each virus type. All source data are provided as a Source Data file.

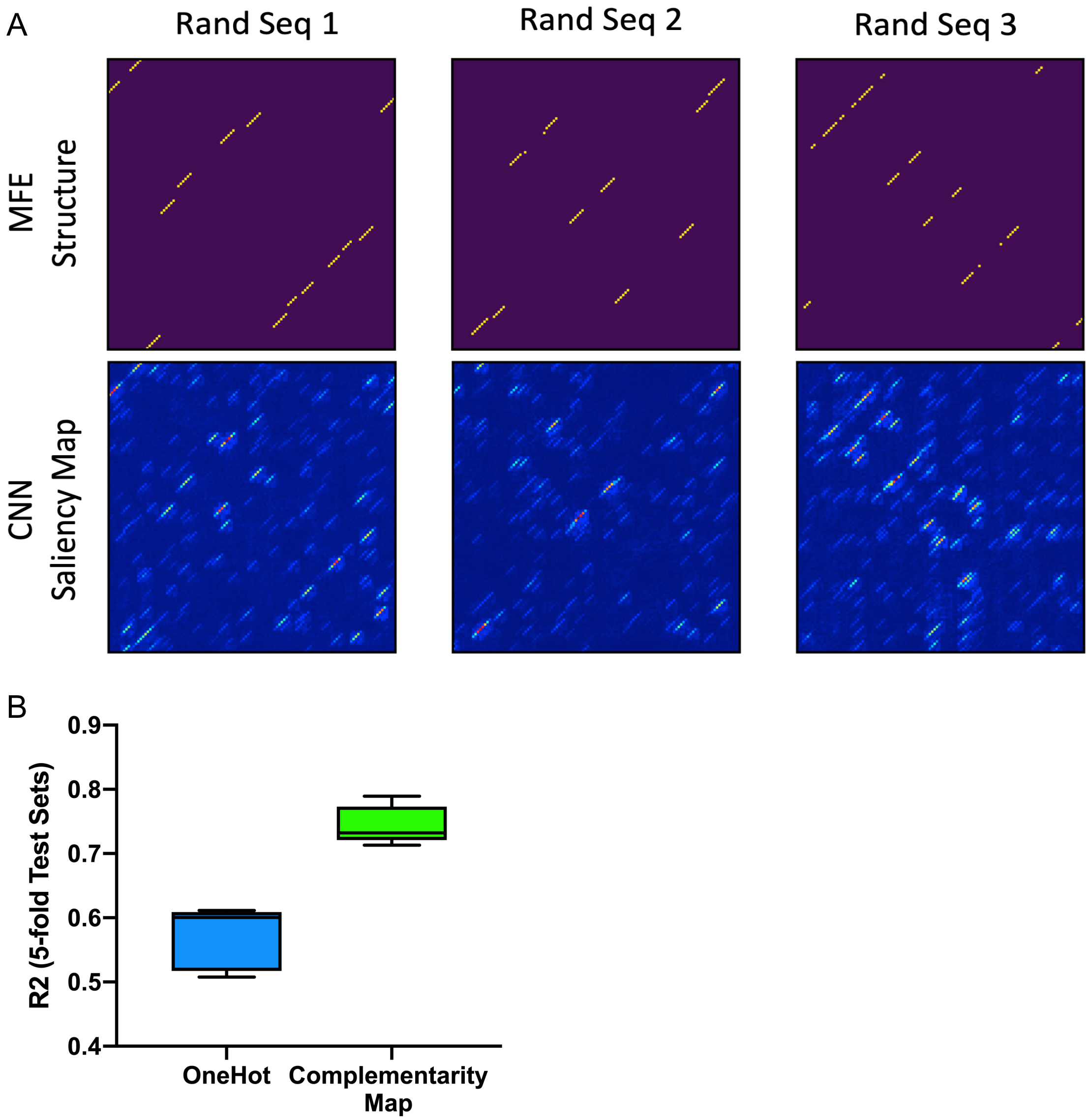

Fig. S11. VIS4Map analysis of random toehold sequences in MFE predictor 2D CNN model. A dataset of 50,000 random RNA sequences of length 120nt and their corresponding MFE values were generated using NUPACK. A convolutional neural network (CNN) was then trained to predict the MFE of each sequence using either a one-hot representation or a complementarity map representation of the sequence as input. (A) For three randomly selected RNA sequences, representative saliency maps generated from the CNN model are shown alongside the MFE structure pre-computed independently using NUPACK. The CNN model was trained on complementarity map inputs. Overlap between salient diagonal features in the VIS4Map outputs and MFE structure maps is visible. (B) We then compared the R^2 coefficients between NUPACK-calculated MFE values and the predictions of a CNN model trained either on a one-hot representation or a complementarity matrix representation of the random RNA sequences. Box and whisker plots summarize n=5 shuffled test sets. Horizontal line indicates the median, box edges are at the 25th and 75th percentiles, and whiskers indicate the smaller of either 1.5× IQR or max/min. All source data are provided as a Source Data file.

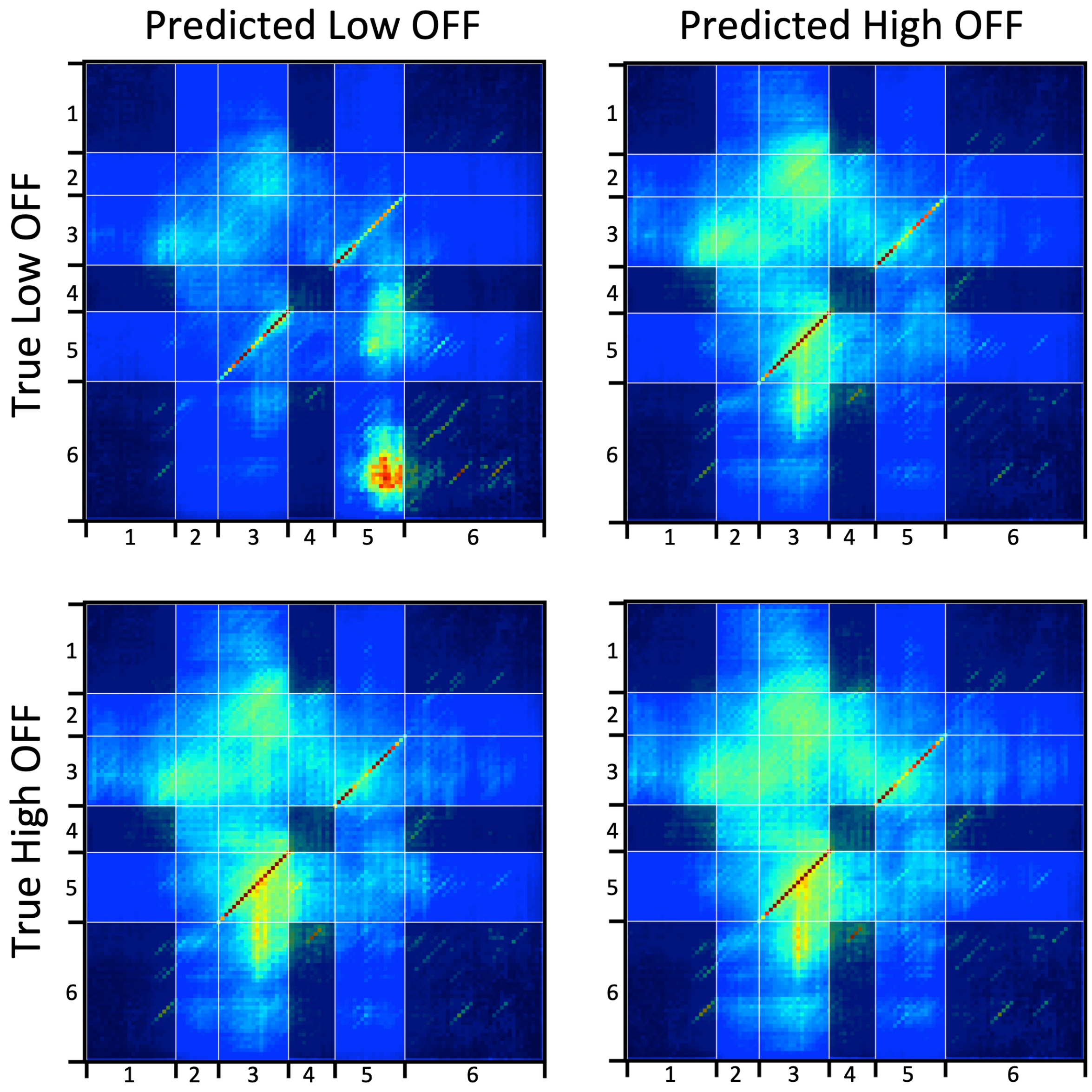

Fig. S12. VIS4Map confusion matrix analysis of the switch OFF conformation. Saliency maps generated from a CNN model trained to predict the toehold switch OFF metric are shown for different ground-truth OFF metrics. The model was trained using a complementarity matrix representation of the toehold sequence as input. Regions labeled on the axes are as follows: (1) constant loop, (2) toehold, (3) ascending stem, (4) constant RBS loop, (5) descending stem, and (6) constant linker. Regions of interaction between constant regions are shaded darker as they do not contain variability between different switch sequences. All saliency maps were generated from the test set only. Saliency maps were then sorted according to the 25% highest and 25% lowest experimentally-determined OFF signal. The 10% best-predicted and 10% worst-predicted saliency maps from the high OFF and low OFF groups were then averaged to produce the shown confusion matrix. Contrast was enhanced four-fold in the averaged maps in order to visualize more sparsely distributed features.

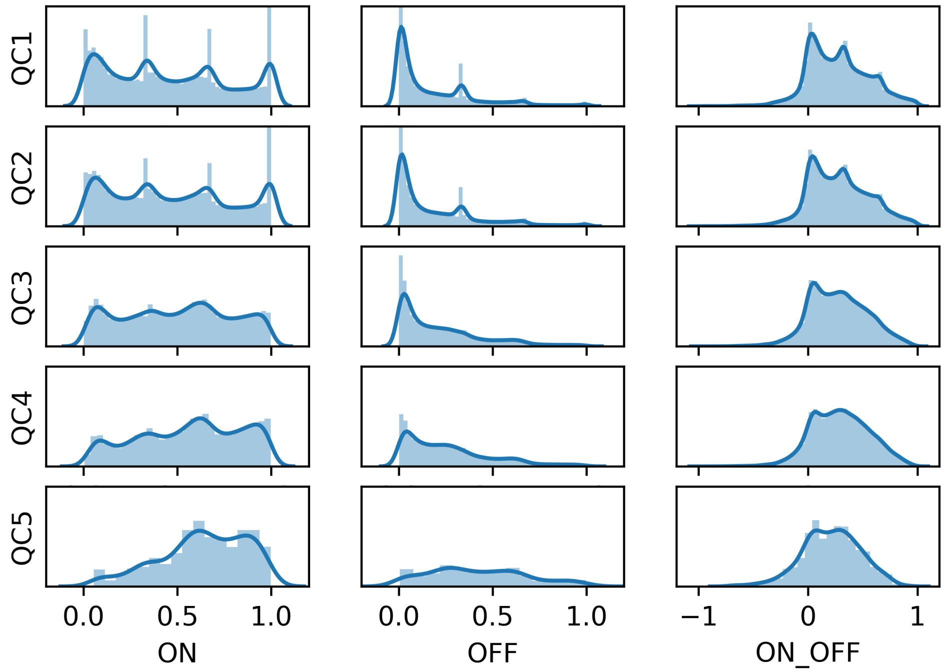

Fig. S13. Dataset distribution vs. QC level. Histograms of toehold switch library values for ON, OFF, and ON/OFF were grouped according to our five different QC threshold levels and are shown here for comparison. The y-axis limits are held constant for ON, OFF, and ON/OFF, respectively, across QC levels after normalizing for data subset size.

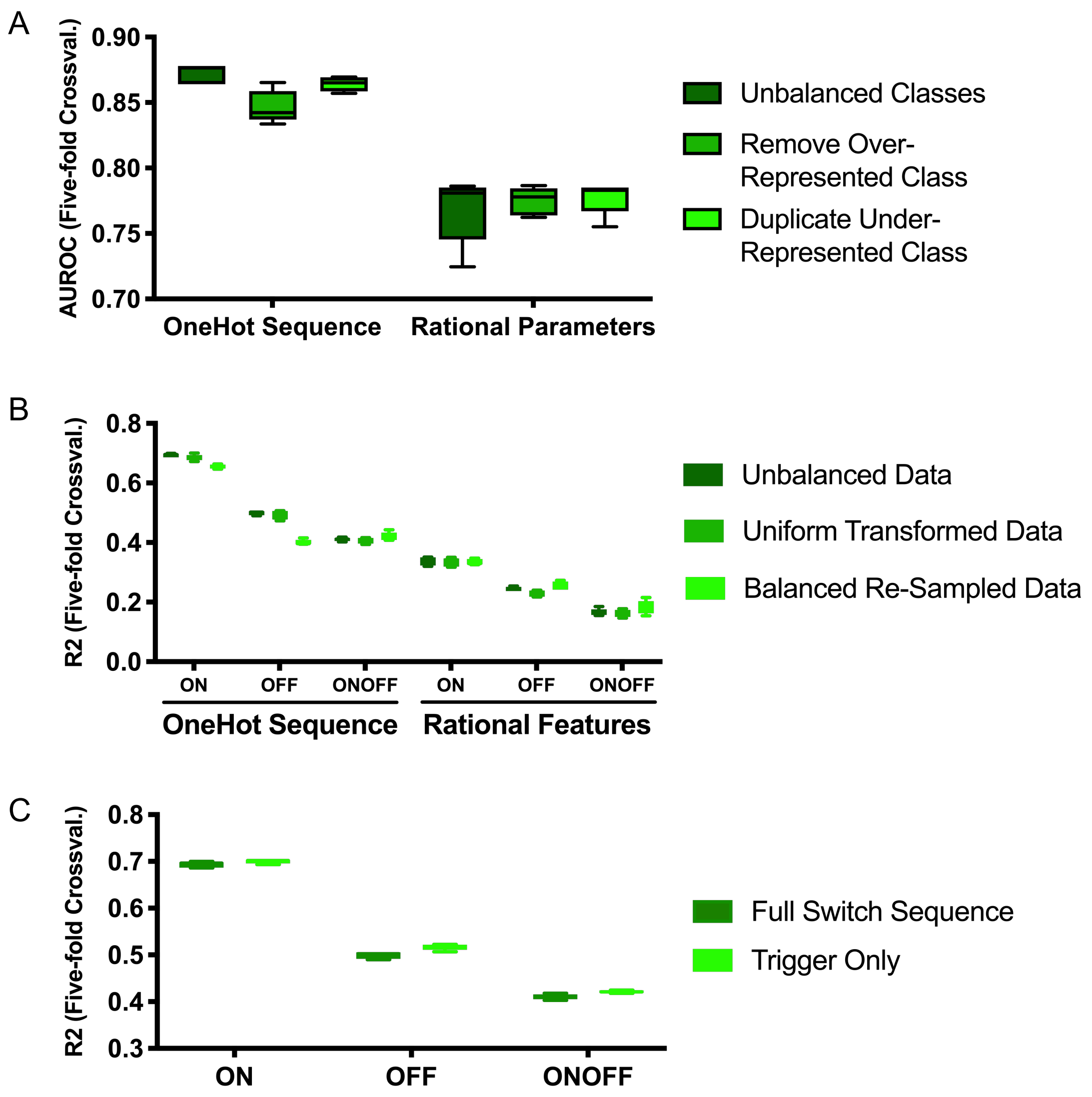

Fig. S14. The effects of categorical label balancing, continuous label balancing, and input sequence length on MLP performance. An MLP was trained on a onehot sequence representation of the toehold switch, and the effect of a variety of input and output scaling parameters were evaluated. (A) Categorical labels for ON/OFF (threshold 0.7) were predicted using either unbalanced data, data balanced by randomly removing data points from the over-represented class, or data balanced by randomly duplicating data points from the under-represented class. The accuracy was measured by calculating the area under the receiver-operator curve (AUROC). (B) Continuous labels for ON, OFF, and ON/OFF were predicted using either raw unbalanced data, data transformed into a uniform distribution using a function that preserves rank-order, or data balanced by randomly re-sampling under-represented values until a uniform distribution was achieved. The accuracy was measured using the R^2 correlation metric (see "Data Balancing" for method details and Supplementary Figure 15 for scatter plots). (C) Continuous labels for ON, OFF, and ON/OFF were predicted while training on the full switch sequence (148nt), or only the trigger sequence (30nt). The accuracy was measured using the R^2 correlation metric (see Supplementary Table 4 for details on subsequences). Box and whisker plots summarize the results of n=5 independently shuffled test sets. Horizontal lines indicate the median, box edges are at the 25th and 75th percentiles, and whiskers indicate the smaller of either 1.5× IQR or max/min. All source data are provided as a Source Data file.

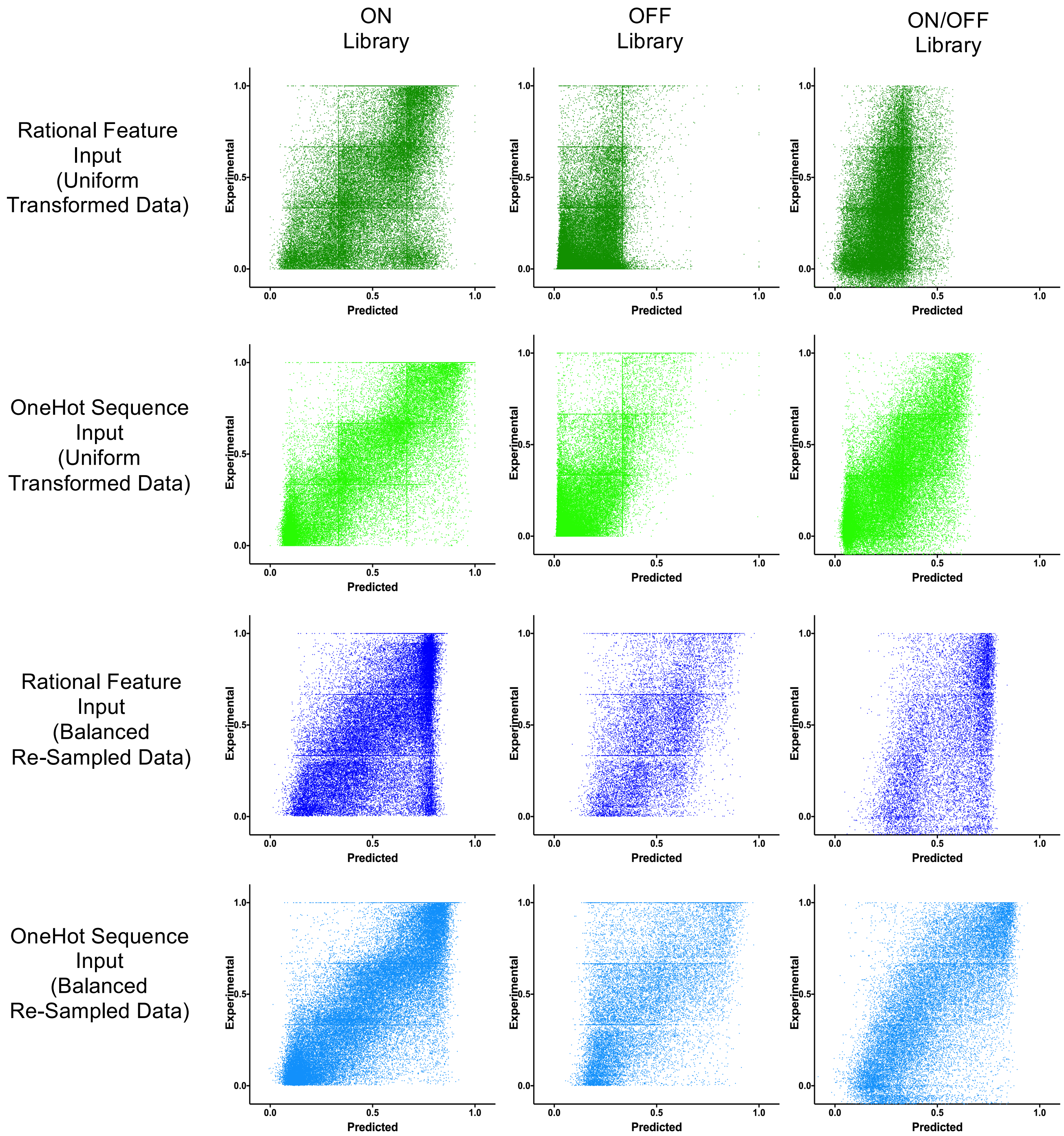

Fig. S15. A comparison of our MLP predictions with our experimental results for uniform and balanced data. Scatter plots of the predicted versus empirical values of compiled test sets are shown for five-fold cross-validated MLP models trained to predict ON, OFF, and ON/OFF using the following input and output data: (i) the rational thermodynamic features as input and uniform-transformed output data (dark green), (ii) the toehold switch one-hot sequence representation as input and uniform-transformed output data (light green), (iii) the rational thermodynamic features as input and balanced re-sampled output data (dark blue), or (iv) the toehold switch one-hot sequence representation as input and balanced re-sampled output data (light blue). The summary statistics are reported in Supplementary Figure 14. See "Data Balancing" for details on the methods used. All source data are provided as a Source Data file.

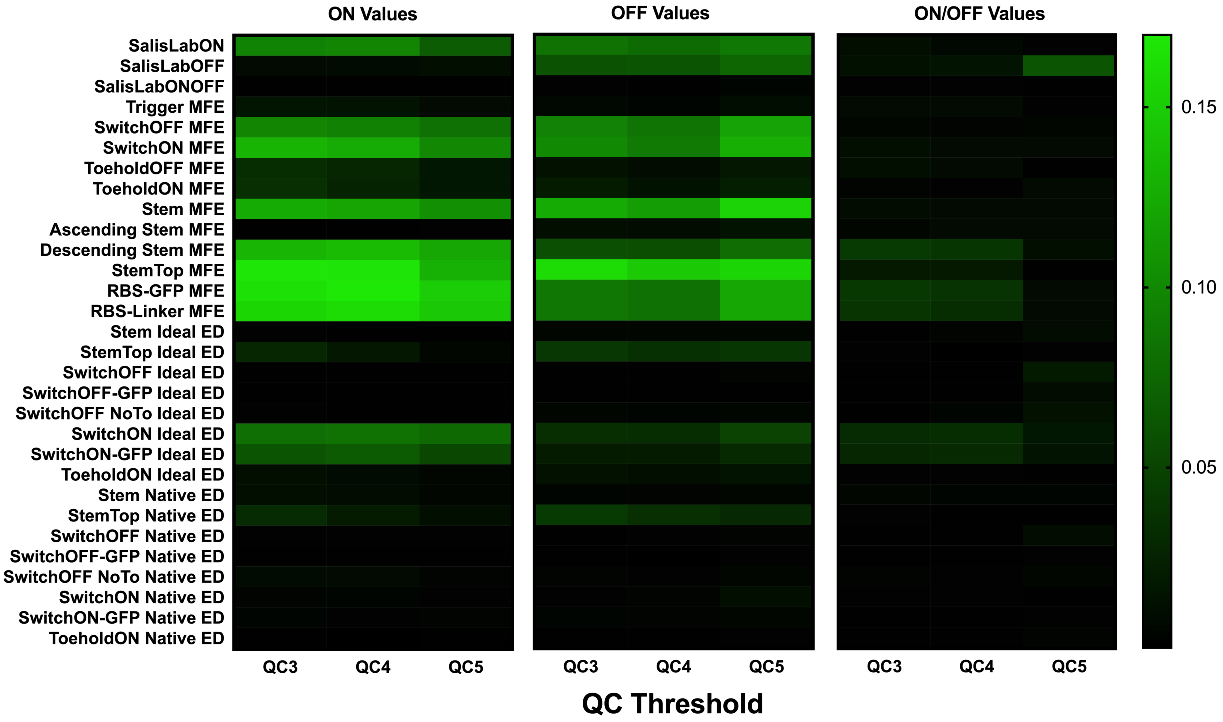

Fig. S16. The effect of QC level on the predictive power of rational thermodynamic features. The R^2 correlations between our dataset and thirty state-of-the-art thermodynamic features as well as RBS Calculator v2.1 outputs, were calculated at higher levels of quality control (QC3, QC4, and QC5 datasets) than are presented in Fig. 3B (calculated for the QC2 dataset). No strong trends in correlation were observed with higher levels of quality control. See Supplementary Table 1 for the conditions for each QC level. All source data are provided as a Source Data file.

	Quality Control Conditions				Library Size		
	OFF Count Threshold	ON Count Threshold	Upper Stdev. Cutoff	Lower Stdev. Cutoff	ON Variants	OFF Variants	ON/OFF Variants
QC1	>= 5	>= 5	None	None	126,620	180,552	110,931
QC2	>= 10	>= 10	None	None	109,067	163,967	91,534
QC3	>= 20	>= 40	None	>0	77,040	90,264	43,044
QC4	>= 60	>= 60	0.4>	>0.04	39,283	67,507	19,983
QC5	>= 300	>= 300	0.4>	>0.04	6,187	12,551	1,137

Table S1. Quality control thresholds. The conditions for inclusion in our five quality control groups (QC1-5) are shown above, including standard deviation cutoffs and library count thresholds. QC2 was ultimately chosen as the final condition for inclusion in our dataset, and all data used or shown in this manuscript is for QC2 unless otherwise stated. The size of each dataset is shown in the three rightmost columns.

	Library #	Trigger Sequence	On	Off
Low 1	1817	CCGACACCTGTTTCATGGAACAATAAAAGA	0.0153	0.0085
Low 2	34792	TGCTGTCTGTGAAACAGATAAATGGAAATA	0.0176	0.0100
Low 3	53587	TCCCTTTCCCAGAAATAAACTTTTTTACCC	0.0181	0.0136
Low 4	72784	TCACTGAGTCATTGCCATCTGCAGAATCAG	0.0048	0.0134
Low 5	104595	TCCAAGACCCAAAGTTCTGGGAACTGGTGG	0.0192	0.0156
Low 6	158538	TGGCAATTGTAGATATAACTTCTGGTAAAT	0.0153	0.0183
Low 7	188705	ATCCAAATATAATGATGACCTATATGCCCT	0.0158	0.0102
Low 8	206071	CCAATATGAGATCTGTAATGCTAACAGTTT	0.0076	0.0146
High 1	79874	GTCATATAAAGGAAGAAGATAGGAGAAGAA	0.9860	0.0031
High 2	111242	AGTTCACAAGAGATGGTTCATGGTGTTCCA	0.9937	0.0132
High 3	158916	AAAGGTTAGCTTATGTTACATATCAAGATA	0.9740	0.0016
High 4	164714	AATCACTGAAAATTGGAGTTAGGTATTGAC	0.9747	0.0007
High 5	166671	GGTATGTTAAGTATGAGGCCTTATCCGTAC	0.9895	0.0115
High 6	187264	TCAAGTTAGAGAAGGAAGTGGCTGAGACCC	0.9856	0.0122
High 7	215129	TAAATCTATGAGAGATCAACGAAAAGGAAG	0.9942	0.0150
High 8	232933	AAAGAAGAAATCATGCAAGAAAACAAAGGG	0.9744	0.0007

Table S2. Toehold switch sequences validated in cell-free format. Sequences of the individually cloned toehold switches for cell-free validation using PURExpress were selected from the QC3 threshold. Their trigger sequences and flow-seq assay performances are shown (see Figure 1F, Supplementary Figure 4 for cell-free assay performance). All highly-functional switches have ON/OFF of 0.97 or greater, while all poorly-functional switches have ON/OFF of 0.04 or less.

ON Triggers	Motif	Counts in Foreground	Counts in Background	P-value	E-value
**Low versus High Signal**					
	UCUYUCU *	349	0	7.10E-122	8.30E-117
	GAUGG	260	19	6.80E-63	7.90E-58
	AAAAA	391	128	1.90E-42	2.10E-37
	CUCYUC *	142	4	1.30E-39	1.40E-34
	UAUUAAC	123	0	1.70E-39	1.90E-34
	UCUCAC *	26	2	4.10E-37	4.50E-32
	GAGUCGU	100	0	5.80E-32	6.30E-27
	GUUUUAUC	100	2	8.50E-29	9.10E-24
**High versus Low Signal**					
	ANSA	785	427	6.00E-62	1.00E-56
	AWUB	644	359	9.50E-38	7.80E-33
	UAYR	355	163	3.90E-23	1.70E-18
	GVRA	270	128	8.20E-16	2.50E-11
	ACK	344	224	1.60E-09	3.80E-05
	AUAA	104	47	8.30E-07	1.40E-02
**OFF Triggers**	**Motif**	**Counts in Foreground**	**Counts in Background**	**P-value**	**E-value**
**Low versus High Signal**					
	CNG	762	503	8.40E-34	1.50E-28
	GRS	510	342	1.90E-14	1.80E-09
	CCUH	218	132	2.60E-07	1.60E-02
**High versus Low Signal**					
	AWWWU	591	346	2.10E-28	3.60E-23
	WUAW	472	333	1.40E-10	1.60E-05
	AAAARA	67	22	5.60E-07	4.30E-02

Table S3. K-mer search results. K-mer motifs searched with DREME (3) using the trigger RNA sequences of the highest and lowest performing 1000 switches sorted by either ON or OFF signal. A one-tailed uncorrected Fisher's Exact Test was used to determine the P-value, and the E-value was further derived from this by correcting for multiple hypotheses. For this search, QC3 dataset was selected. * Denotes potential anti-SD pyrimidine-rich sequences.

Rational Feature Sub-sequence Name	Sequence Region	Brief Description
SwitchOFF	30-108	Toehold switch off conformation
SwitchOFF-GFP	30-144	Off conformation with added GFP sequence
SwitchOFF-NoTo	62-144	Off conformation with toehold removed
SwitchON	0-108	Toehold switch on conformation
SwitchON-GFP	0-144	On conformation with added GFP sequence
Trigger	0-29	Trigger sequence alone
ToeholdOFF	30-62	Toehold region of switch including link1
ToeholdON	0-62	Toehold region only hybridized to trigger
Stem	62-108	Stem only of toehold switch
AscendingStem	62-100	Ascending arm of the switch stem
DescendingStem	80-108	Descending arm of the switch stem
StemTop	74-97	Top half of the stem from start codon up
RBS-Linker	80-134	Region from RBS loop2 to linker
RBS-GFP	80-144	RBS-Linker with added GFP sequence

**[-3,-1]**	**[0,29]**	**[30,49]**	**[50,79]**	**[80,90]**	**[91,96]**	**[97,99]**	**[100,108]**	**[109,134]**	**[135,144]**
GGG	trigger	loop1	switch	loop2	stem1	AUG	stem2	linker	post-linker

Table S4. Rational feature sub-sequences. The sub-sequences from which the 30 rational features used as MLP input were calculated using ViennaRNA are shown here in the upper panel. In the lower panel, we show the full un-truncated toehold switch sequence framework from which the sub-sequences in the top table were selected.

Primer Name	Primer Sequence	Primer Purpose
ColE1 Backbone F	cctcaggcatttgagaagcacacgGcaacgaaagccagatagcccgtac	Used to transfer toehold switch to ColE1 backbone
ColE1 Backbone R	gcgacagttagcccagagcagcGgctgaaaggaggaactatatccgg	Used to transfer toehold switch to ColE1 backbone
ColE1 Insert F	ccggatatagttcctcctttcagcCgctgctctgggctaactgtcgc	Used to transfer toehold switch to ColE1 backbone
ColE1 Insert R	tgtacgggctatctggctttcgttgCcgtgtgcttctcaaatgcctgagg	Used to transfer toehold switch to ColE1 backbone
ColEI Del BsmBI F1	tcactggtgaaaagaaaaaccaccctgg	Used to delete an undesired BsmBI restriction site from ColE1 Backbone
ColEI Del BsmBI R1	atccgggagctgcatgtgtcagagg	Used to delete an undesired BsmBI restriction site from ColE1 Backbone
ColEI Del BsmBI F2	acggtcacagcttgtctgtaagcggatg	Used to delete an undesired BsmBI restriction site from ColE1 Backbone
ColEI Del BsmBI R2	tacgggcaacagctgattgccc	Used to delete an undesired BsmBI restriction site from ColE1 Backbone
TH Library Ins F	TCCGGATATAGTTCCTCCTTTCAG	Used to amplify Toehold Switch Inserts post-sorting for NGS
TH Library Ins R	AGTGAAAAGTTCTTCTCCTTTACGC	Used to amplify Toehold Switch Inserts post-sorting for NGS
Trig 4	AATTGATATTGTGATTATGTGATGATTGTACCCTATAGTGAGTCGTATTAGCGC	Used produce Green et al trigger RNA
Trig 10	AATTGATATTGTTCGTTTCGTATGATCTAACCCTATAGTGAGTCGTATTAGCGC	Used produce Green et al trigger RNA
Trig 14	AATTGATATTGTAGTATGTTGAAGTGATTGCCCTATAGTGAGTCGTATTAGCGC	Used produce Green et al trigger RNA
Trig 42	AATTGATATTGTTAGTGTTATAGGCGTTAGCCCTATAGTGAGTCGTATTAGCGC	Used produce Green et al trigger RNA
Trig 46	AATTGATATTGTGCTGTTTATGTGCGTTCGCCCTATAGTGAGTCGTATTAGCGC	Used produce Green et al trigger RNA
Trig 48	CTCATTATCTATAGTTCGTCGAGGGTCTTACCCTATAGTGAGTCGTATTAGCGC	Used produce Green et al trigger RNA
Trig 55	AATGATATGTGTAGTTCGTCGAGGTGTCCACCCTATAGTGAGTCGTATTAGCGC	Used produce Green et al trigger RNA
Trig 56	ATAATGTAAGTAAGTTCGTCGAGGTGTCCACCCTATAGTGAGTCGTATTAGCGC	Used produce Green et al trigger RNA
Trig 59	AATTGATATTGTTAGTAGTGTATGATTCGGCCCTATAGTGAGTCGTATTAGCGC	Used produce Green et al trigger RNA
Trig 63	AATTGATATTGTAGGTTTCTGATGCGCTTACCCTATAGTGAGTCGTATTAGCGC	Used produce Green et al trigger RNA
Trig 64	TACAAGATATAGAGTTCGTCGAGGCTTAGACCCTATAGTGAGTCGTATTAGCGC	Used produce Green et al trigger RNA
Trig 68	AATGTATGTAATAGTTCGTCGAGGTGTCCACCCTATAGTGAGTCGTATTAGCGC	Used produce Green et al trigger RNA
Trig 70	AATTGATATTGTAGTAGTTGTATGTGCGCGCCCTATAGTGAGTCGTATTAGCGC	Used produce Green et al trigger RNA
Trig 88	AATTGATATTGTGCTAGTGTTATGATTCTGCCCTATAGTGAGTCGTATTAGCGC	Used produce Green et al trigger RNA
Trig 101	TTATTCCTGTATAGTTCGTCGAGGTGTCCACCCTATAGTGAGTCGTATTAGCGC	Used produce Green et al trigger RNA
Trig 112	ATCTTGTATTGTAGTTCGTCGAGGGTATGACCCTATAGTGAGTCGTATTAGCGC	Used produce Green et al trigger RNA
Trig 117	TCAATAAGGCGGAGTTCGTCGAGGTGCCTGCCCTATAGTGAGTCGTATTAGCGC	Used produce Green et al trigger RNA
Trig 134	AATTGATATTGTTCGTATGTTATGTCGCCGCCCTATAGTGAGTCGTATTAGCGC	Used produce Green et al trigger RNA
Trig 145	AATTGATATTGTGAAGTTAGGATGGTAGTGCCCTATAGTGAGTCGTATTAGCGC	Used produce Green et al trigger RNA
Trig 159	CGTATATCATTAAGTTCGTCGAGGTCCGTGCCCTATAGTGAGTCGTATTAGCGC	Used produce Green et al trigger RNA
Switch 4	GCGCTAATACGACTCACTATAGGGAATTGATATTGTGATTATGTGATGATTGTAAACAGAGGAGATACAATATGCACATAATCAACCTGGCGGCAGCGCAAAAGATGCG	Used to clone Green et al switch

Switch 10	GCGCTAATACGACTCACTATAGGGAATTGATATTGTTCGTTTCGT<br>ATGATCTAAGACAGAGGAGATTAGATATGACGAAACGAAACCTGG<br>CGGCAGCGCAAAAGATGCG	Used to clone Green et al switch
Switch 14	GCGCTAATACGACTCACTATAGGGAATTGATATTGTAGTATGTTG<br>AAGTGATTGAACAGAGGAGACAATCAATGCAACATACTAACCTGG<br>CGGCAGCGCAAAAGATGCG	Used to clone Green et al switch
Switch 42	GCGCTAATACGACTCACTATAGGGAATTGATATTGTTAGTGTTAT<br>AGGCGTTAGAACAGAGGAGACTAACGATGATAACACTAAACCTGG<br>CGGCAGCGCAAAAGATGCG	Used to clone Green et al switch
Switch 46	GCGCTAATACGACTCACTATAGGGAATTGATATTGTGCTGTTTAT<br>GTGCGTTCGGACAGAGGAGACGAACGATGATAAACAGCAACCTGG<br>CGGCAGCGCAAAAGATGCG	Used to clone Green et al switch
Switch 48	GCGCTAATACGACTCACTATAGGGCTCATTATCTATAGTTCGTCG<br>AGGGTCTTAAGCAGAGGAGATAAGACATGCGACGAACTAACCTGG<br>CGGCAGCGCAAAAGATGCG	Used to clone Green et al switch
Switch 55	GCGCTAATACGACTCACTATAGGGAATGATATGTGTAGTTCGTCG<br>AGGTGTCCAAGCAGAGGAGATGGACAATGCGACGAACTAACCTGG<br>CGGCAGCGCAAAAGATGCG	Used to clone Green et al switch
Switch 56	GCGCTAATACGACTCACTATAGGGATAATGTAAGTAAGTTCGTCG<br>AGGTGTCCAAGCAGAGGAGATGGACAATGCGACGAACTAACCTGG<br>CGGCAGCGCAAAAGATGCG	Used to clone Green et al switch
Switch 59	GCGCTAATACGACTCACTATAGGGAATTGATATTGTTAGTAGTGT<br>ATGATTCGGAACAGAGGAGACCGAATATGACACTACTAAACCTGG<br>CGGCAGCGCAAAAGATGCG	Used to clone Green et al switch
Switch 63	GCGCTAATACGACTCACTATAGGGAATTGATATTGTAGGTTTCTG<br>ATGCGCTTAAACAGAGGAGATAAGCGATGCAGAAACCTAACCTGG<br>CGGCAGCGCAAAAGATGCG	Used to clone Green et al switch
Switch 64	GCGCTAATACGACTCACTATAGGGTACAAGATATAGAGTTCGTCG<br>AGGCTTAGAAGCAGAGGAGATCTAAGATGCGACGAACTAACCTGG<br>CGGCAGCGCAAAAGATGCG	Used to clone Green et al switch
Switch 68	GCGCTAATACGACTCACTATAGGGAATGTATGTAATAGTTCGTCG<br>AGGTGTCCAAGCAGAGGAGATGGACAATGCGACGAACTAACCTGG<br>CGGCAGCGCAAAAGATGCG	Used to clone Green et al switch
Switch 70	GCGCTAATACGACTCACTATAGGGAATTGATATTGTAGTAGTTGT<br>ATGTGCGCGAACAGAGGAGACGCGCAATGACAACTACTAACCTGG<br>CGGCAGCGCAAAAGATGCG	Used to clone Green et al switch
Switch 88	GCGCTAATACGACTCACTATAGGGAATTGATATTGTGCTAGTGTT<br>ATGATTCTGGACAGAGGAGACAGAATATGAACACTAGCAACCTGG<br>CGGCAGCGCAAAAGATGCG	Used to clone Green et al switch
Switch 101	GCGCTAATACGACTCACTATAGGGTTATTCCTGTATAGTTCGTCG<br>AGGTGTCCAAGCAGAGGAGATGGACAATGCGACGAACTAACCTGG<br>CGGCAGCGCAAAAGATGCG	Used to clone Green et al switch
Switch 112	GCGCTAATACGACTCACTATAGGGATCTTGTATTGTAGTTCGTCG<br>AGGGTATGAAGCAGAGGAGATCATACATGCGACGAACTAACCTGG<br>CGGCAGCGCAAAAGATGCG	Used to clone Green et al switch
Switch 117	GCGCTAATACGACTCACTATAGGGTCAATAAGGCGGAGTTCGTCG<br>AGGTGCCTGAGCAGAGGAGACAGGCAATGCGACGAACTAACCTGG<br>CGGCAGCGCAAAAGATGCG	Used to clone Green et al switch
Switch 134	GCGCTAATACGACTCACTATAGGGAATTGATATTGTTCGTATGTT<br>ATGTCGCCGAACAGAGGAGACGGCGAATGAACATACGAAACCTGG<br>CGGCAGCGCAAAAGATGCG	Used to clone Green et al switch
Switch 145	GCGCTAATACGACTCACTATAGGGAATTGATATTGTGAAGTTAGG<br>ATGGTAGTGAACAGAGGAGACACTACATGCCTAACTTCAACCTGG<br>CGGCAGCGCAAAAGATGCG	Used to clone Green et al switch
Switch 159	GCGCTAATACGACTCACTATAGGGCGTATATCATTAAGTTCGTCG<br>AGGTCCGTGAGCAGAGGAGACACGGAATGCGACGAACTAACCTGG<br>CGGCAGCGCAAAAGATGCG	Used to clone Green et al switch

Table S5. Primers used in this study. A list of all primers used to create the data reported in this work are listed, including their sequence, name, and primary purpose.

Supplementary References

1. A. A. Green, P. A. Silver, J. J. Collins, P. Yin, Toehold switches: de-novo-designed regulators of gene expression. *Cell* **159**, 925-939 (2014).
2. A. Espah Borujeni, H. M. Salis, Translation initiation is controlled by RNA folding kinetics via a ribosome drafting mechanism. *Journal of the American Chemical Society* **138**, 7016-7023 (2016).
3. T. L. Bailey, DREME: motif discovery in transcription factor ChIP-seq data. *Bioinformatics* **27**, 1653-1659 (2011).
