# Supplementary material for: A deep learning approach to programmable RNA switches
Source: Nat Commun. 2020 Oct 7;11:5057. doi: 10.1038/s41467-020-18677-1 (PMC7541447; doi:10.1038/s41467-020-18677-1)
Supplement: Supplementary file 3 — Reporting Summary [file 41467_2020_18677_MOESM3_ESM.pdf]

nature research

Corresponding author(s): Jim Collins

Last updated by author(s): July 27, 2020

Reporting Summary

Nature Research wishes to improve the reproducibility of the work that we publish. This form provides structure for consistency and transparency in reporting. For further information on Nature Research policies, see our Editorial Policies and the Editorial Policy Checklist.

Statistics

For all statistical analyses, confirm that the following items are present in the figure legend, table legend, main text, or Methods section.

n/a	Confirmed	
☐	☒	The exact sample size (*n*) for each experimental group/condition, given as a discrete number and unit of measurement
☐	☒	A statement on whether measurements were taken from distinct samples or whether the same sample was measured repeatedly
☐	☒	The statistical test(s) used AND whether they are one- or two-sided<br>*Only common tests should be described solely by name; describe more complex techniques in the Methods section.*
☐	☒	A description of all covariates tested
☐	☒	A description of any assumptions or corrections, such as tests of normality and adjustment for multiple comparisons
☐	☒	A full description of the statistical parameters including central tendency (e.g. means) or other basic estimates (e.g. regression coefficient) AND variation (e.g. standard deviation) or associated estimates of uncertainty (e.g. confidence intervals)
☐	☒	For null hypothesis testing, the test statistic (e.g. *F*, *t*, *r*) with confidence intervals, effect sizes, degrees of freedom and *P* value noted<br>*Give P values as exact values whenever suitable.*
☐	☒	For Bayesian analysis, information on the choice of priors and Markov chain Monte Carlo settings
☐	☒	For hierarchical and complex designs, identification of the appropriate level for tests and full reporting of outcomes
☐	☒	Estimates of effect sizes (e.g. Cohen's *d*, Pearson's *r*), indicating how they were calculated

Our web collection on statistics for biologists contains articles on many of the points above.

Software and code

Policy information about availability of computer code

Data collection	Raw paired-end sequencing reads from an Illumina NextSeq instrument were quality filtered and merged with PEAR 0.9.1.
Data analysis	All code and analysis was performed in Python 3.6 using the following libraries: seaborn 0.9, matplotlib 3.0.2, ViennaRNA 2.4.11 (aka NUPACK), TensorFlow 1.10.0, Keras 2.2.0, Sony Sorter Software SH800S, and DREME 5.1.1. Code is available at https://github.com/lrsoenksen/CL_RNA_SynthBio

For manuscripts utilizing custom algorithms or software that are central to the research but not yet described in published literature, software must be made available to editors and reviewers. We strongly encourage code deposition in a community repository (e.g. GitHub). See the Nature Research guidelines for submitting code & software for further information.

Data

Policy information about availability of data

All manuscripts must include a data availability statement. This statement should provide the following information, where applicable:

- Accession codes, unique identifiers, or web links for publicly available datasets
- A list of figures that have associated raw data
- A description of any restrictions on data availability

All custom code used in this work, including that used to train and test deep learning models, perform saliency visualizations, and perform ViennaRNA/Nupack/Kinfold calculations, can be obtained from the following publicly accessible GitHub page:

https://github.com/lrsoenksen/CL_RNA_SynthBio

A .csv file containing the complete toehold switch dataset is also available from the same GitHub page, which includes read counts for each sorting gate, derived

flow-seq data, assigned QC scores, switch subsequences, and calculated rational parameter values. The same dataset as well as raw NGS seq read data can be obtained using GEO accession GSE149225. Raw source data for figures 2A-2F, 3B, 3D-3G, 4A-4D, 5B, and supplementary figures 1B-1E, 2A-2B, 3, 4, 5, 6, 7A-7F, 8A-8C, 9, 10A-10D, 11B, 14A-14C, 15, 16, are provided with the paper.

Field-specific reporting

Please select the one below that is the best fit for your research. If you are not sure, read the appropriate sections before making your selection.

- [x] Life sciences
- [ ] Behavioural & social sciences
- [ ] Ecological, evolutionary & environmental sciences

For a reference copy of the document with all sections, see nature.com/documents/nr-reporting-summary-flat.pdf

Life sciences study design

All studies must disclose on these points even when the disclosure is negative.

Sample size	An optimal cutoff threshold for the number of NGS reads required for a given switch variant to be included in the dataset was determined based on the accuracy of a onehot-sequence multilayer perceptron (MLP) model, as well as the R2 correlation and MAE between measurements in the dataset and a smaller replicate dataset. At a cutoff of 10 reads (QC process #2), a high level of correlation was observed between our two replicate datasets as measured with the R2 metric and MAE, and in addition the performance in training and testing of the MLP model ceased to improve past this threshold, suggesting that the sample size was sufficient to effectively estimate the population mean.
Data exclusions	NGS reads corresponding to incorrectly synthesized switches were omitted from the dataset.
Replication	A replicate dataset was produced under identical conditions for library transformation, growth, induction, sorting, and NGS data processing. While smaller, this replicate dataset showed a high degree of correlation with our main dataset.
Randomization	Because each switch is induced in its own individual, single E. coli cell, each measurement taken is perfectly independent and randomized with respect to other measurements.
Blinding	The researchers were not blinded to the contents of NGS pools. Due to the pooled nature of the flow-seq assay, measurements of individual toehold switches within the overall population could not be affected by the behavior of the researchers (the individual switches being handled all together in bulk), and to facilitate practicalities relating to the proper assignment of NGS barcodes for data processing, blinding was not used.

Reporting for specific materials, systems and methods

We require information from authors about some types of materials, experimental systems and methods used in many studies. Here, indicate whether each material, system or method listed is relevant to your study. If you are not sure if a list item applies to your research, read the appropriate section before selecting a response.

Materials & experimental systems

n/a	Involved in the study	
[x]	[ ]	Antibodies
[x]	[ ]	Eukaryotic cell lines
[x]	[ ]	Palaeontology and archaeology
[x]	[ ]	Animals and other organisms
[x]	[ ]	Human research participants
[x]	[ ]	Clinical data
[x]	[ ]	Dual use research of concern

Methods

n/a	Involved in the study	
[x]	[ ]	ChIP-seq
[ ]	[x]	Flow cytometry
[x]	[ ]	MRI-based neuroimaging

Flow Cytometry

Plots

Confirm that:

- [x] The axis labels state the marker and fluorochrome used (e.g. CD4-FITC).
- [x] The axis scales are clearly visible. Include numbers along axes only for bottom left plot of group (a 'group' is an analysis of identical markers).
- [x] All plots are contour plots with outliers or pseudocolor plots.
- [x] A numerical value for number of cells or percentage (with statistics) is provided.

Methodology

Sample preparation	BL21 E. coli cells were induced with IPTG and grown in LB broth at 37C prior to flow sorting.
Instrument	Sony SH800 FACS machine.
Software	Sony Cell Sorter Software (SH800S).
Cell population abundance	All sorted fractions contained at least 10% of the total population of events (at least 10 million cells), and the purity of E. coli cells collected was ensured with antibiotic selection during subsequent growth.
Gating strategy	Positive and negative control plasmids were used to define the sorting boundaries prior to sorting of the library.

☒ Tick this box to confirm that a figure exemplifying the gating strategy is provided in the Supplementary Information.
